# (Re) Making the Procrustean Bed? Standardization and Customization as Competing Logics in Healthcare

**DOI:** 10.15171/ijhpm.2017.35

**Published:** 2017-03-28

**Authors:** Russell Mannion, Mark Exworthy

**Affiliations:** Health Services Management Centre, University of Birmingham, Birmingham, UK.

**Keywords:** Standardization, Customization, Personalization, Competing Logics

## Abstract

Recent years have witnessed a parallel and seemingly contradictory trend towards both the standardization and**** the customization of healthcare and medical treatment. Here, we explore what is meant by ‘standardization’ and ‘customization’ in healthcare settings and explore the implications of these changes for healthcare delivery. We frame the paradox of these divergent and opposing factors in terms of institutional logics – the socially constructed rules, practices and beliefs which perpetuate institutional behaviour. As the tension between standardization and customization is fast becoming a critical fault-line within many health systems, there remains an urgent need for more sustained work exploring how these competing logics are articulated, adapted, resisted and co-exist on the front line of care delivery.

## On Myths, Magic and Murder

According to ancient Greek mythology, there once lived in Attica an evil inn-keeper named Damastes who was nicknamed Procrustes, or ‘the Stretcher.’ Feigning hospitality to weary pilgrims, he would offer them a ‘magic bed’ for the night. The bed was made of iron, and he liked to boast that the length of the bed was always an exact match for anyone who lay down upon it. After his guests were plied with ample food and drink, he would tie them to the bed. There was never an exact fit, as Procrustes secretly possessed two beds. If they were too long he would amputate their limbs in order to fit the bed. If they were too short he would place them on a rack and stretch them until they fitted the dimensions of the bed. This ‘customization’ of the *guest* to the *bed* mattered little since, in either event the victim died.

Metaphorically, the Procrustean bed represents an arbitrary standardization to which conformity is forced, and it is not difficult to find parallels in modern healthcare where standards and standardization are ubiquitous. But whereas standards are typically deemed laudatory, as something to aspire to, standardization has been associated with a bland “one size fits all” approach which privileges rationality at the expense of humanity. Recent years have also witnessed a parallel (and seemingly contradictory) trend towards the customization and personalization of health and medical care. In this editorial, we unpack what is meant by ‘standardization’ and ‘customization’ in healthcare and explore some of the implications for healthcare delivery. We frame the paradox of these apparently divergent and opposing factors in terms of institutional logics – the socially constructed rules, practices and beliefs which perpetuate the institution.^1^ This, we argue, helps to explain better the motives, meanings and impact of this interplay between standardization and customization in modern healthcare.

## On Standardizing Complexity?

It has been claimed that healthcare is undergoing a process known as the “industrialization of medicine” which comprises three-sequential phases: (*i*) *formalization*; (*ii*) *standardization;* (*iii*) and *automation*, with contemporary health systems currently at the second stage.^2^ *Standardization* prescribes or limits behaviour and procedures, and has been defined formally as “the process of developing, agreeing upon and implementing uniform technical specifications, criteria, methods, processes, designs or practices that can increase compatibility, interoperability, safety, repeatability and quality.”^3^ Standardization facilitates commensurability and therefore comparison between services, clinicians and organization.^4^ As a result, standardization (together with quantification and competition) is seen to be part of a neo-liberal accountability.^5^

For our purposes, Timmermans and Epstein^6^ usefully distinguish between four sub-types of standards which underpin standardization processes:

- *Design standards* are detailed specifications which explicitly define the properties and features of products or services to ensure uniformity and compatibility. In healthcare an obvious example is the promulgation of standardized clinical practice guidelines (see discussion below).
- *Terminological standards* (such as The International Classification of Disease) support stability of meaning and help coordinate dispersed actions across cultures, distances and time.
- *Performance standards* set outcome specifications for service delivery. For example, maximum waiting times that are deemed as acceptable for access to hospital care.
- *Procedural standards* specify how processes are to be performed and the steps to be taken. For example, in the development of formal Service Frameworks used in disease management programmes.

For each, it is helpful to interpret this typology in terms of the ways in which these standards are interpreted and enacted in institutions by agents.

In the field of healthcare, the standardization of uniform healthcare treatment reached its apotheosis in the evidence-based medicine (EBM) movement. In particular, the promotion of clinical practice guidelines whereby experts evaluate the best available evidence and on the basis of this, guidelines, protocols and checklists are designed to standardize procedures in the expectation that this is the best way to reduce unwanted variation in diagnosis and treatment.^7,8^ This formalized nature of EBM was supposed to reduce variations in practice that had previously been hidden by public deference (which presumed an equality of competence among clinicians) and a lack of information.^9^ Standardization has become a function of risk-based systems which, in turn, has spawned consequent auditing processes to assure such risk is managed.^10^ This has had particular resonance in healthcare through initiatives such as clinical governance and the proliferation of bureaucratic rules, clinical protocols, hygiene procedures and surgical checklists which attempt to create order by standardizing organizational behavior and professional practice.^8,11^ However, the assumed logic of EBM has come under sustained criticism by social scientists.^12^ Evidence-based standards are, on occasion, not founded on the “best” evidence but on opinion and consensus, which is seen to be weaker in quality.^13^ Meta-reviews have shown, for example, that only in 50% of cases do clinicians follow clinical practice guidelines endorsed by national and professional medical organizations. In an ethnographic study in UK primary care, it was found that general practitioner (GP) clinicians only rarely consult clinical guidelines directly when making clinical decisions.^14^ Rather, they prefer to rely on what have been termed ‘mindlines’ – collectively reinforced, internalised tacit guidelines – which are informed by a brief reading, but mainly by their interactions with colleagues and patients and by other sources of largely tacit knowledge that built on their early training and experience. This underlines the need to consider the ways in which institutional agents interpret and enact standardization in their own context.

Moreover, there appears to be varying acceptance and resistance to standards and standardization among different health professionals^15^ with some professional groups viewing guidelines as undermining their expertise and autonomy.^16^ For example, McDonald and colleagues^17^ explored the contrasting and conflicting world views of managers and clinicians towards quality and patient safety. They found that managers firmly believed that standardized linear solutions based on adherence to guidelines would lead to beneficial improvements in patient safety. In contrast, the medical perspective was opposed to the standardisation of clinical practice and advocated the legitimacy of professional judgement and the toleration of uncertainty and risk. In essence, doctors believed that patient safety was an art or craft whereas managers approached it as a science.^18^ It is clear that health professionals employ a range of strategies and behaviours in order to ‘get the job done’ and sidestep ‘problematic’ rules and guidelines. Such behaviours circumvent workflow blocks and have variously been termed workarounds^19^; violations^20^ and shortcuts.^21^ Workarounds undermine standardization and organizationally prescribed procedures and have been shown, in different contexts, to contributing towards either subverting or augmenting patient safety.^19^

In a McDonaldized world of healthcare standardization, the dominant logic is for each patient/burger to be processed in exactly the same prescribed way irrespective of individual values and preferences with ethical behaviour interpreted as compliance to (external) institutional rules and standards. Ethical action then becomes loyalty to the organization (or profession) rather than the patient and being moral becomes procedural.^22^ At the extreme, an excessive focus on meeting external performance standards may lead to catastrophic failings in professional practice, as graphically illustrated by recent hospital scandals and lapses in professional practice in the English National Health Service (NHS).^23,24^

## On the Customization, Personalization and the Individualization of Healthcare

Customization would appear to be in direct opposition to standardization.^25^ Whilst we consider personalization and individualization to fall under the title of customization, there are subtle differences between them. For example, healthcare has, to some extent, always been individualized, reflecting the supposed infinite variety of conditions expressed through patients and thus the indeterminacy of care.

Unprecedented scientific breakthroughs and technological advancements, not least the completion of the Human Genome Project and the advancement of pharmogenomics, has heralded a new era of customized and personalized healthcare and medicine. This has been accelerated by the emergence of a more commodified and consumerist approach to health and healthcare. Four dominant interventions and approaches to customization that have gained traction across diverse health systems can be discerned:

First, healthcare management systems are becoming more customized in the sense that they allow better delivery of highly individualised interventions (through prediction, prevention and treatment) which are tailored to an individual’s unique genetic, physiological or psychological characteristics.^26^ Personalized medicine introduces the ability to use molecular markers that signal disease risk before symptoms appear, and it offers the opportunity to focus on prevention and early intervention rather than on reaction at advanced stages of disease. Such prognostication would thus supersede the biomedical treatment model of healthcare.

Second, healthcare services and treatments that are personalized in the sense that they seek to treat each individual holistically as a ‘whole person’ and which are respectful of a patient’s particular values, wishes and lifestyle (including the preference not to take responsibility for their own care). For example, in many health systems, there has been a shift towards more shared decision making processes in which patients are involved as active partners with health professionals in clarifying acceptable medical options and choosing a preferred course of care and treatment.^27^

Third, management or treatment that is individualised in the sense that healthcare is provided as a good or commodity with consumers expressing their choices and wants via demand side signals in the market. Patient choice is promoted on the basis that it creates competition between providers thereby leading to improvements in efficiency and quality. But the evidence on this is rather mixed.^28^

Fourth, health and medical care is personalised in the sense that more responsibility for the management of healthcare is taken on by individuals themselves or their carers rather than healthcare professionals. The introduction of personal budgets in the NHS is an example of devolving control over purchasing decision to patients who act as commissioners of their own care. Furthermore, alliances between patient and clinicians to ‘co-produce’ care has arisen from the growing expertise of patients, especially those with chronic conditions.^29^

These developments towards more customization and personalization bring their own tensions and problems, not least the increased obligations and expectations on individuals to take an active role in their own care and wellbeing. The process by which patients (and even the wider public) become responsible for their own health transforms some of them into ‘calculating selves’ - any failure to act (to improve their health, for example) thus becomes their own responsibility.^30^ Yet, personal characteristics can affect the extent to which individuals want or are able to engage in their health and care. These include an individual’s social and cultural background, their health status and their beliefs and preferences as well as social determinants of health. It also may mean that people come to feel guilt and anxiety if they do not fulfil the expectations placed on them to be active participants in their health and care. Personalisation may also undermine the solidarity of welfare systems (especially healthcare) as it isolates more clearly the benefits and contributions.^31^

## On Researching Standardization and Customization

Ostensibly, standardization and customization present sharply differing ways of organizing and delivering healthcare. How might we explain these divergent and opposing trends? One such way is using the notion of institutional logics. These are “the socially constructed, historical pattern of material practices, assumptions, values, beliefs, and rules by which individuals produce and reproduce their material subsistence, organize time and space, and provide meaning to their social reality.”^32^ Although the rise of standardization and more recently customization can be discerned as macro-trends in health system reform, it is at the meso (organizational) and micro level (team and individual) that such logics play out and impact on professional practice and patient care.^33^ The articulation between such levels presents an opportunity for in-depth research into the meanings of and interactions between actors at these levels. Moreover, such levels of standardization and customisation are, in practice, rarely achieved and indeed, often meld into hybrid forms.^34^ So, in other industries (such as car manufacturing, for example), we observe forms of ‘mass customization’ whereby organizations are able to reap the benefits of standardization whilst simultaneously ensuring individual/local adaptations.^35^ This is increasingly aided by new production and delivery processes as well as applying the power of ‘big data.’ We may also observe, however, other competing logics; two which have been subject to significant attention are professionalism and managerialism.^36^

In some ways, such an addition sediments logics upon logics,^37^ and so leads to greater complexity to be described and explained.^38^ Institutional pillars is one framework to understand the articulation between standardization and customization, and how they might compete as rival logics. Scott argues that: “Institutions are comprised of regulative, normative, and cultural-cognitive elements that, together with associated activities and resources, provide stability and meaning to social life” (p.48).^38^ The regulative pillar refers to rule-setting, the normative pillar to obligations and the cultural pillar to symbolic systems; in others words, to what must be done, what should be done and what is done.

An alternative, theoretical, framework is that of local universality.^15^ Here it is argued that universality merges “localized processes of negotiation” (p.275); that is, standardization of, say, protocols needs to be rooted in existing work practices at the same time that those practices are transformed by the protocol. This is a tension between incorporation and transformation, and has echoes of Dugdale’s notion of the convergence from ‘sameness’ to ‘difference.’^39^ A key tenet of such work, and which we have demonstrated earlier, is the absence of a central actor, coordinating processes of standardization (or equally, customization).

Although there is a small but growing body of research exploring continuity and change in healthcare organizations through the prism of an institutional logics perspective,^40,41^ there remains an urgent need for more sustained work exploring how the competing logics of standardization and customization are articulated, adapted, blended and/or resisted on the front line of care delivery^36^ and indeed how such logics interact and contribute towards (re)making (or dismantling) the Procrustean bed in modern healthcare. As the tension between standardization and customization is fast becoming a critical fault-line within many health systems, the need for such work becomes even more compelling.

## Ethical issues

Not applicable.

## Competing interests

Authors declare that they have no competing interests.

## Authors’ contributions

Both authors contributed equally to the writing of this article.
